# Reproducibility of frequency-dependent low frequency fluctuations in reaction time over time and across tasks

**DOI:** 10.1371/journal.pone.0184476

**Published:** 2017-09-14

**Authors:** Zan-Zan Liu, Hui-Jie Qu, Zhuo-Ling Tian, Meng-Jian Han, Yi Fan, Lie-Zhong Ge, Yu-Feng Zang, Hang Zhang

**Affiliations:** 1 Department of Psychology, Zhejiang Sci-Tech University, Hangzhou, China; 2 Institutes of Psychological Sciences, Hangzhou Normal University, Hangzhou, China; 3 Zhejiang Key Laboratory for Research in Assessment of Cognitive Impairments, Hangzhou, China; 4 Center for Cognition and Brain Disorders, Hangzhou Normal University, Hangzhou, China; 5 Department of Physics, Zhejiang Sci-Tech University, Hangzhou, China; 6 Paul C. Lauterbur Research Centers for Biomedical Imaging, Shenzhen Institutes of Advanced Technology, Chinese Academy of Sciences, Shenzhen, China; Universita degli Studi di Palermo, ITALY

## Abstract

**Objective:**

Increased levels of reaction time variability (RTV) are characteristics of sustained attention deficits. The clinical significance of RTV has been widely recognized. However, the reliability of RTV measurements has not been widely studied. The present study aimed to assess the test-retest reliability of RTV conventional measurements, e.g., the standard deviation (SD), the coefficient of variation (CV), and a new measurement called the amplitude of low frequency fluctuation (ALFF) of RT. In addition, we aimed to assess differences and similarities of these measurements between different tasks.

**Method:**

Thirty-seven healthy college students participated in 2 tasks, i.e., an Eriksen flanker task (EFT) and a simple reaction task (SRT), twice over a mean interval of 56 days. Conventional measurements of RTV including RT-SD and RT-CV were assessed first. Then the RT time series were converted into frequency domains, and RT-ALFF was further calculated for the whole frequency band (0.0023–0.167 Hz) and for a few sub-frequency bands including Slow-6 (<0.01 Hz), Slow-5 (0.01–0.027 Hz), Slow-4 (0.027–0.073 Hz), and Slow-3 (0.073–0.167 Hz). The test-retest reliability of these measurements was evaluated through intra-class correlation (ICC) tests. Differences and correlations between each EFT and SRT measurement were further examined during both visits.

**Results:**

1) The RT-ALFF of the Slow-5/4/3 and conventional measurements of RT-SD and RT-CV showed moderate to high levels of test-retest reliability. EFT RT-ALFF patterns generated slightly higher ICC values than SRT values in higher frequency bands (Slow-3), but SRT RT-ALFF values showed slightly higher ICC values than EFT values in lower frequency bands (Slow-5 and Slow-4). 2) RT-ALFF magnitudes in each sub-frequency band were greater for the SRT than those for the EFT. 3) The RT-ALFF in the Slow-4 of the EFT was found to be correlated with the RT-ALFF in the Slow-5 of the SRT for both two visits, but no consistently significant correlation was found between the same frequency bands.

**Conclusions:**

These findings reveal good test-retest reliability for conventional measurements and for the RT-ALFF of RTV. The RT-ALFF presented frequency-dependent similarities across tasks. All of our results reveal the presence of different frequency structures between the two tasks, and thus the frequency-dependent characteristics of different tasks deserve more attention in future studies.

## 1. Introduction

Reaction time variability (RTV) refers to variations in an individual’s response speed across trials during engagement in a stimulus-response task. RTV is believed to be more than just noise, and elevated RTV levels have been taken as a representation of the occurrence of attention lapses [1–3]. Clinically, RTV has been proposed to be a potential endophenotype of several mental disorders involving inattentive symptoms, e.g., attention deficit hyperactivity disorder (ADHD) [4–6], bipolar disorder [7] and traumatic brain injury (TBI) [8].

Widely used measurements of individual RTV include the standard deviation (SD) and coefficient of variation (CV) [9]. However, neither of these can depict periodic fluctuations in RT over time. Castellanos and colleagues (2005) were the first to convert the time course of the RT of the Eriksen flanker task (EFT) into frequency domains and to then identify periodic fluctuations in RT [10]. Their results indicate that RT fluctuations reach a peak amplitude of approximately 0.05 Hz and importantly that the peak amplitude of the RT of children with ADHD is significantly higher than that of controls [10]. Such results have been replicated in several studies [11, 12]. These studies show that RT fluctuations present increased amplitudes in the frequency band of 0.01–0.1 Hz for disorders involving inattentive symptoms. Intriguingly, the frequency band of 0.01–0.1 Hz is considered to be a critical frequency band of brain activity according to resting-state functional magnetic resonance imaging (RS-fMRI) studies [13, 14]. The amplitude of low frequency fluctuation (ALFF) is widely used in RS-fMRI studies to characterize fluctuations in spontaneous brain activity [15]. Thus, this paper borrows the term ALFF to depict RT fluctuations. Recent studies have investigated the RT-ALFF patterns [11, 16, 17] of several sub-frequency bands (e.g., Slow-6 (< 0.01 Hz), Slow-5 (0.01–0.027 Hz), Slow-4 (0.027–0.073 Hz) and Slow-3 (0.073–0.198 Hz)). These sub-frequency bands are defined by Penttonen and Buzsaki based on specific properties and physiological functions [18]. These studies show that RT-ALFF in the Slow-4 can better distinguish individuals with ADHD from controls than other sub-frequency bands [11], and it has been observed that RT-ALFF in the Slow-4 can predict attention problems in a large general population sample [19]. RT-ALFF patterns of inattentive symptoms may be frequency-dependent [11].

The clinical significance of RTV has been widely recognized [4–8] for a number of decades. However, the test-retest reliability of such measurements of RTV has not yet been well studied. The measurement of test-retest reliability levels is fundamental to any new metric of behavioral measurement or psychological testing [20]. To date, many results of sustained attention have been generated from conventional and new RTV measurements. Nevertheless, the test-retest reliability of such measurements has not been clearly elucidated, and such issues are central to understanding previous findings.

RTV is typically measured through the application of different cognitive tasks, and such tasks mostly vary in complexity and cognitive demand. The EFT, as a widely used task in RT-ALFF studies, is relatively complex. It involves three conditions, i.e., neutral, congruent, and incongruent. Thus, cognitive processes of the EFT recruit at least selection and inhibition in addition to sustained attention. Moreover, the mean RT values of the three conditions vary significantly. Differences in RT between conditions inevitably contribute to the total RTV value, a widely used measurement of sustained attention. In fact, sustained attention should be detected through a simple and repetitive task [21]. The simple reaction task (SRT) is likely the simplest reaction time task. The SRT involves very limited levels of selection and inhibition processing and is simple and monotonous. The complexity and cognitive demands of the EFT and SRT are quite different, but both tasks involve sustained attention. Sustained attention is a primary ability of an individual. Therefore, measurements of sustained attention might show reproducibility across tasks. It will be interesting to determine how much sustained attention measurements of the two tasks are similar or different.

The present study aimed to investigate the reproducibility of RTV measurements over time and across tasks. Two tasks (EFT and SRT) were employed. RTV measurements mainly included the following: RT-SD, RT-CV, and RT-ALFF. Three issues were addressed: 1) the test-retest reliability of these measurements; 2) measurement differences between the EFT and SRT; and 3) measurement correlations between the two tasks.

## 2. Method

### 2.1. Participants

Thirty-seven healthy college students (18 females, *M*_age_ = 21 ± 2 years, ranging from 18 to 27 years of age) were recruited to participate in our study. All of the participants were right-handed, and no one reported any history of brain injury or mental disorders. Each participant signed an informed consent form before the experiment was conducted. The study was approved by the Center for Cognition and Brain Disorders (CCBD) Ethics Committee of Hangzhou Normal University.

### 2.2. Experimental procedure

The experimental procedure involved two visits conducted over a mean interval of 56 days (ranging from 54 to 58 days). For each visit, two tasks (EFT and SRT) were performed. The order in which the two tasks were conducted was counter-balanced across the participants. Tasks were programmed in E-prime 1.1 (Psychology Software Tools, Inc.).

**Fig 1:**
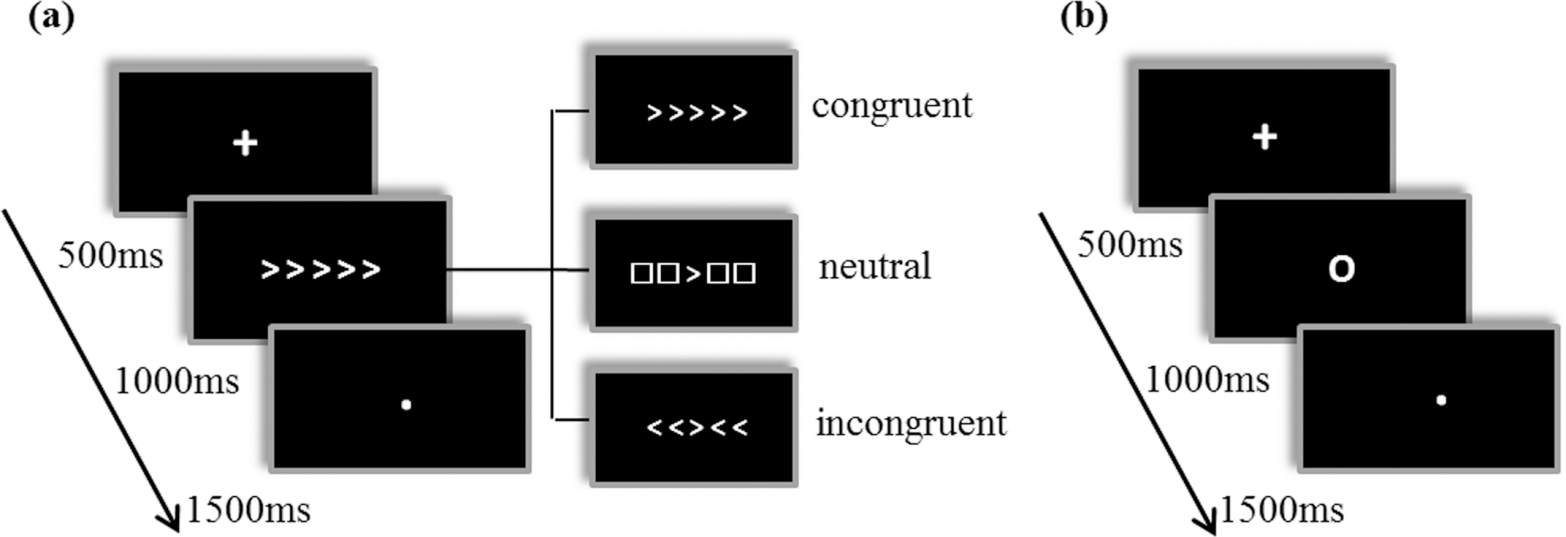
Experimental procedures. (a) The Eriksen flanker task (EFT) procedure; (b) the simple reaction task (SRT) procedure.

We employed an arrow version of EFT [22] through which three conditions (neutral, congruent, and incongruent) were presented in pseudorandom order with equal probability (Fig 1A). Trials were presented continuously with a fixed 3-s inter-trial interval (ITI) to allow for a frequency analysis. At the start of each trial, a warning signal (a fixation cross) was displayed in the middle of the screen (500 ms). The stimulus was then presented (1,000 ms) horizontally. Each participant was instructed to determine the direction (left or right) of the middle arrow (Fig 1A) and to press “F” or “J” on the computer keyboard with one of his or her index fingers as accurately and quickly as possible. A fixation dot was then displayed (1,500 ms). The task involved 156 trials, and we administered the same pseudorandom sequence of trials to all participants. Before the formal task was completed, we employed one or two 30-trial blocks to ensure that the participants achieved an accuracy reading of at least 80% during the test.

The SRT was adapted from a previous study [23]. To perform a frequency-dependent analysis, we employed a fixed ITI (3 s). For each trial, a fixation cross (500 ms) was presented followed by a target “O” (1,000 ms) and then a fixation dot (1,500 ms) (Fig 1B). We employed 156 trials in total. Each participant was instructed to press the “J” key with his or her right index finger when the target was displayed. Prior to this task, the participants completed a short practice round to achieve an accuracy reading of at least 80%.

### 2.3. Data analysis

#### 2.3.1. Pre-processing and measurement

The first 10 trials (30 s) of the 156 trial tasks were discarded, as the participants were still growing accustomed to the task during these trials. Participants presenting extreme mean RT values (longer than three SDs beyond the mean RT for all of the participants) were excluded from further analysis. Two participants were excluded according to this criterion, and 35 participants were studied further.

Conventional measurements (the RT-mean, RT-SD, RT-CV, omission error rate, commission error rate, and directional error rate (for EFT only) were first taken for each visit. Then, the new measurement (RT-ALFF) was taken over the following steps: i) Missing and anticipatory responses (RT≤100 ms) were interpolated by linear interpolation [24] to reconstruct an integrated time series. ii) RT data were standardized by dividing by the mean RT to render the amplitude between the two tasks comparable. iii) Linear trends were removed. iv) A power spectrum was obtained from the Fast Fourier Transform (FFT). v) RT-ALFF was calculated in sub-frequency bands Slow-6 (< 0.01 Hz), Slow-5 (0.01–0.027 Hz), Slow-4 (0.027–0.073 Hz), and Slow-3 (0.073–0.167 Hz) following from many previous behavioral [11, 16, 17] and RS-fMRI studies [25, 26]. Here, RT-ALFF was defined as the square root of the power spectrum following from the measure employed in Di Martino et al. [11]. The RT-ALFF of the whole frequency band (0.0023–0.167 Hz) was also calculated. The ITI of the EFT and SRT was measured as 3 s and thus the frequency band examinable was 0.0023–0.167 Hz according to Nyquist’s sampling theorem.

#### 2.3.2. Statistical analysis

The EFT involved three conditions, i.e., congruent, neutral, and incongruent, and conventional measurements could be taken based on each condition. Thus, differences in these measurements, i.e., RT-mean, RT-SD and RT-CV, across the conditions were examined first. The repeated measure of ANOVA was applied to each measurement with two factors, i.e., condition (three levels) and visit (two levels). Differences in each measurement between the conditions were then tested for each visit.

An analysis of test-retest reliability was performed on the EFT and SRT. For each measurement, the intra-class correlation (ICC) coefficient [27] between the first and second visits was calculated. For RT-ALFF measurements, ICC calculations were based on each frequency band.

To explore measurement differences between the tasks, a paired-*t* test was performed on RT-mean, RT-SD and RT-CV values derived from the two tasks. Wilcoxon signed rank tests, which are nonparametric, were applied to error rates. For RT-ALFF levels, differences between the two tasks were investigated for each visit by applying a repeated ANOVA with two within-subject factors (tasks and frequency bands).

The correlations of each measurement between tasks were assessed for each visit. The Pearson correlation of each conventional measurement between the EFT and SRT was assessed. For RT-ALFF levels, a RT-ALFF correlation analysis between tasks was performed for each sub-frequency band. Notably, error rates were not normally distributed, and thus the test-retest reliability of each task and the correlation of error rates between each task were evaluated from the Spearman correlation.

## 3. Results

### 3.1. General EFT results

The values of conventional measurements, i.e., RT-mean, RT-SD and RT-CV, for the three conditions of the EFT are depicted in Table 1. The RT-mean was found to be the longest for the incongruent condition and the shortest for the congruent condition consistent with previous studies [28, 29] (see S1 Table).

**Table 1:** Conventional measurements of the Eriksen flanker task (EFT) for three conditions.

	congruent	neutral	incongruent
	*Mean* (*SD*)	*Mean* (*SD*)	*Mean* (*SD*)
**First Visit**			
RT-mean (ms)	471 (45)	480 (50)	529 (51)
RT-SD (ms)	52 (16)	53 (17)	63 (22)
RT-CV (%)	10.95 (2.76)	11.02 (2.93)	11.81 (3.44)
**Second Visit**			
RT-mean (ms)	467 (40)	474 (40)	520 (46)
RT-SD (ms)	51 (15)	51 (17)	61 (20)
RT-CV (%)	10.69 (2.71)	10.59 (3.11)	11.62 (3.28)

### 3.2. Test-retest reliability of the measurements

As applied in previous studies [30, 31], the ICC value was categorized into five levels: poor (ICC < 0.2), fair (0.2 < ICC < 0.39), moderate (0.4 < ICC < 0.59), high (0.6 < ICC < 0.79), and excellent (0.8 < ICC < 1). The test-retest reliability of the conventional measurements, i.e., RT-mean, RT-SD, and RT-CV, and error rates are shown in Table 2. The reliability of the RT-mean was found to be excellent for both tasks. Moderate to excellent levels of reliability were found for RT-mean, RT-SD, and RT-CV values but not for error rates. No significant difference in these conventional measurements was found between the two visits (all |*t*| (34) < 1.217, *p* > .05, all |*Z* (34)| < 1.34, *p* > .05).

**Table 2:** Test-retest reliability of conventional measurements of the Eriksen flanker task (EFT) and simple reaction task (SRT). Intra-class correlation (ICC) coefficients were calculated for RT-mean, RT-SD, and RT-CV values. Spearman correlation coefficients (*r*) were calculated for error rates.

	First Visit		Second Visit			
	*Mean*	*SD*	*Mean*	*SD*	ICC / *r*	*p*
**EFT**						
RT-mean (ms)	492	48	486	40	.84*	< .001
RT-SD (ms)	63	15	60	16	.77*	< .001
RT-CV (%)	12.71	2.45	12.28	2.62	.70*	< .001
Omission error rate (%)	0.10	0.29	0.08	0.28	.26	.14
Commission error rate (%)	0	0	0	0	NA	NA
Directional error rate (%)	1.14	1.60	0.92	0.91	.30	.08
**SRT**						
RT-mean (ms)	319	68	315	67	.84*	< .001
RT-SD (ms)	71	19	70	20	.61*	.004
RT-CV (%)	22.50	5.30	22.55	6.34	.53	.016
Omission error rate (%)	0.98	1.88	0.67	1.25	.25	.14
Commission error rate (%)	1.33	2.52	0.96	1.81	.37	.028

* denotes corrected *p* < 0.05 (for EFT, *p* < 0.008 for six comparisons; for SRT, *p* < 0.01 for five comparisons).

ICC values of RT-ALFF between the two visits are shown in Table 3. The ICC of RT-ALFF for the whole frequency band (0.0023–0.167 Hz) was found to be high for the EFT (ICC (34) = .69) and moderate for the SRT (ICC (34) = .59). For the sub-frequency bands, both tasks presented moderate to high levels of RT-ALFF test-retest reliability for the Slow-5 (0.01–0.027 Hz), Slow-4 (0.027–0.073 Hz) and Slow-3 (0.073–0.167 Hz). The ICC of RT-ALFF for the lowest sub-frequency band (Slow-6) was found to be poor for both tasks.

**Table 3:** Intra-class correlation (ICC) coefficients of RT-ALFF between iterations of the Eriksen flanker task (EFT) and simple reaction task (SRT).

	First Visit		Second Visit			
	*Mean*	*SD*	*Mean*	*SD*	ICC	*P*
**EFT**						
Slow-6 (<0.01 Hz)	1.64	0.51	1.59	0.55	- 0.29	.76
Slow-5 (0.01–0.027 Hz)	1.52	0.49	1.40	0.34	.53	.014
Slow-4 (0.027–0.073 Hz)	1.36	0.28	1.30	0.32	.56*	.009
Slow-3 (0.073–0.167 Hz)	1.28	0.27	1.26	0.27	.74*	< .001
Whole band	1.33	0.27	1.29	0.27	.69*	.001
**SRT**						
Slow-6 (<0.01 Hz)	3.77	1.65	3.18	1.19	.15	.30
Slow-5 (0.01–0.027 Hz)	2.74	0.69	2.77	0.99	.58*	.008
Slow-4 (0.027–0.073 Hz)	2.36	0.61	2.36	0.75	.68*	.001
Slow-3 (0.073–0.167 Hz)	2.12	0.55	2.14	0.72	.40	.07
Whole band	2.33	0.56	2.31	0.69	.59*	.007

* denotes corrected *p* < .05 (for both EFT and SRT, *p* < .01 for five comparisons).

### 3.3. Differences in measurements between tasks

As was expected, the RT-mean of the EFT was found to be significantly longer than that of the SRT while RT-SD and RT-CV values of the EFT were found to be lower than those of the SRT (see Table 4). In addition, the SRT presented more omission and commission errors than the EFT (corrected *p* < .05 for omission errors of each visit; no commission error was found for the EFT of each visit).

**Table 4:** Comparisons of conventional measurements made between tasks for each visit. A paired-t test (*t*) was performed on RT-mean, RT-SD and RT-CV values. A Wilcoxon signed rank test (*Z*) was performed on omission error rates.

	EFT		SRT			
	*Mean*	*SD*	*Mean*	*SD*	*t*/*Z*	*P*
**First Visit**						
RT-mean (ms)	492	48	319	68	17.97*	< .001
RT-SD (ms)	63	15	71	19	- 1.99	.055
RT-CV (%)	12.71	2.45	22.50	5.30	- 9.94*	< .001
Omission error rate (%)	0.10	0.29	0.98	1.88	- 3.16*	.002
**Second Visit**						
RT-mean (ms)	486	40	315	67	21.43*	< .001
RT-SD (ms)	60	16	70	20	- 2.86*	.007
RT-CV (%)	12.28	2.62	22.55	6.34	-9.73*	< .001
Omission error rate (%)	0.08	0.28	0.67	1.25	- 3.01*	.003

* denotes corrected *p* < .05 (for each visit, *p* < .0125 for four comparisons).

**Fig 2:**
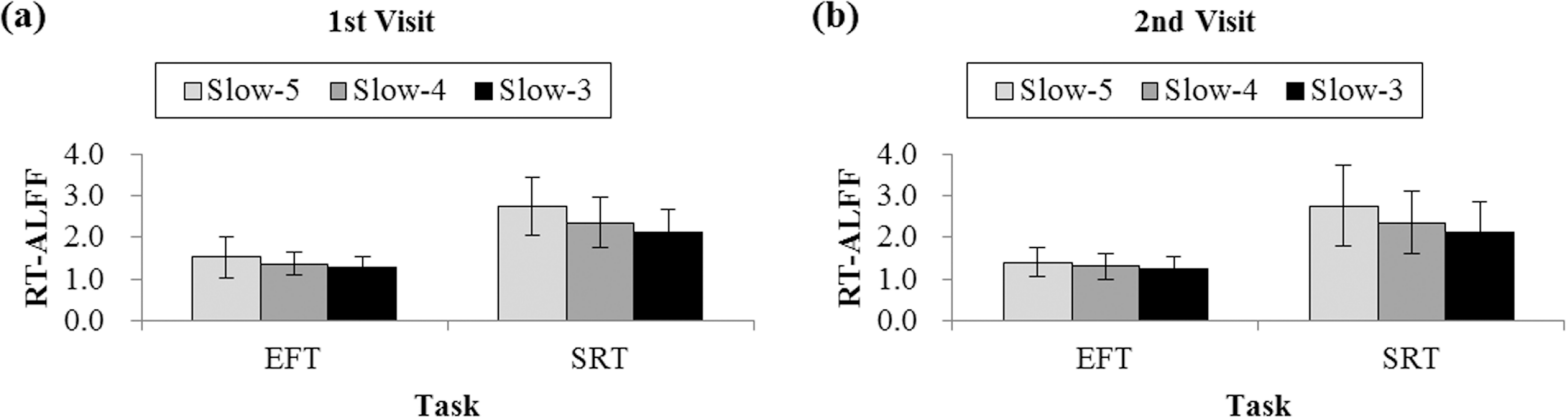
RT-ALFF values for the Slow-5 (0.01–0.027 Hz), Slow-4 (0.027–0.073 Hz) and Slow-3 (0.073–0.167 Hz) of the EFT and SRT. (a) RT-ALFF patterns of the first visit; (b) RT-ALFF patterns of the second visit. For each sub-frequency band, the SRT generated higher RT-ALFF values than the EFT for both visits, and significant Task × Sub-frequency Band interactions were found for both visits. RT-ALFF differences between tasks were found to be the most significant for Slow-5.

Regarding RT-ALFF patterns, the repeated measure of ANOVA with two within-subject factors (Task: two levels, i.e., EFT and SRT; Sub-frequency band: three levels, i.e., Slow-5/4/3) showed a significant main effect of the tasks (Fig 2A and 2B. *F* (1, 34) = 101.66, *p* < .001, η_p_^2^ = .75 for the first visit; *F* (1, 34) = 88.59, *p* < .001, η_p_^2^ = .72 for the second visit) and a main effect of the sub-frequency band (*F* (2, 33) = 29.08, *p* < .001, η_p_^2^ = .64 for the first visit; *F* (2, 33) = 15.99, *p* < .001, η_p_^2^ = .49 for the second visit) for both visits. As is illustrated in Fig 2, the SRT generated a higher RT-ALFF than the EFT for each sub-frequency band, and RT-ALFF levels decreased with increasing frequency levels for both tasks. Significant interactions between sub-frequency bands and tasks were found for both visits (*F* (2, 33) = 6.62, *p* = .004, η_p_^2^ = .29 for the first visit; *F* (2, 33) = 7.23, *p* = .002, η_p_^2^ = .30 for the second visit). A follow-up analysis of each sub-frequency band shows that the SRT generated a significantly higher RT-ALFF than the EFT for each visit (each *t* > 7.17, *p* < .001, corrected *p* < .05) with the largest difference found in the lower frequency band (Slow-5, Fig 2).

### 3.4. Correlations of measurements between tasks

As is shown in Table 5, significant correlations between tasks were only found for the RT-mean. RT-SD correlations for the second visit approached a significant value (*r* (33) = .41, *p* = .014, corrected *α* = .0125 for 4 comparisons, Bonferroni adjustment).

**Table 5:** Correlations (*r*) of conventional measurements between the Eriksen flanker task (EFT) and simple reaction task (SRT) for each visit.

	*r*	*P*
**First Visit**		
RT-mean	.56*	< .001
RT-SD	.02	.92
RT-CV	.01	.98
Omission errors	- 0.12	.49
**Second Visit**		
RT-mean	.72*	< .001
RT-SD	.41	.014
RT-CV	.24	.16
Omission errors	.07	.68

* denotes corrected *p* < .05 (for each visit, *p* < .0125 for four comparisons).

For RT-ALFF patterns, correlations between the two tasks were assessed for each frequency band with the exception of Slow-6 owing to its poor test-retest reliability. Of the 6 correlations of the two visits (3 for each) (Table 6), only Slow-4 for the second visit was found to be significantly correlated, but it was found to be highly inconsistent with that of the first visit (*r* = .49 vs. *r* = .08). As the three conditions (congruent, incongruent, and neutral) of the EFT frequently switch, i.e., in a relatively high frequency manner, the frequency structure of the two tasks can be different. We thus performed a band-shift correlation, i.e., the RT-ALFF correlation for a frequency band of one task with the neighboring frequency band of another task (Table 7). Interestingly, the overall band-shift correlation was found to be very different from the corresponding band correlation (i.e., the same frequency band) (Table 7 vs. Table 6). The two visits showed consistently significant correlations (*r* = .357 for the first visit and *r* = .356 for the second visit; *p* < .05, uncorrected).

**Table 6:** Correlations (*r*) of RT-ALFF between the EFT and SRT for corresponding bands.

	*r*	*P*
**First Visit**		
Slow-5 (0.01–0.027 Hz)	.22	.20
Slow-4 (0.027–0.073 Hz)	.08	.65
Slow-3 (0.073–0.167 Hz)	.04	.81
**Second Visit**		
Slow-5 (0.01–0.027 Hz)	.31	.07
Slow-4 (0.027–0.073 Hz)	.49*	.003
Slow-3 (0.073–0.167 Hz)	.16	.36

* denotes corrected *p* < .05 (for each visit, *p* < .017 for corrections of three comparisons).

**Table 7:** Band-shift correlations (*r*) of RT-ALFF between tasks for each visit.

	*r*	*P*
**First Visit**		
Slow-4 (EFT)–Slow-5 (SRT)	.357*	.035
Slow-3 (EFT)–Slow-4 (SRT)	.01	.94
Slow-5 (EFT)–Slow-4 (SRT)	.22	.20
Slow-4 (EFT)–Slow-3 (SRT)	.08	.67
**Second Visit**		
Slow-4 (EFT)–Slow-5 (SRT)	.356*	.036
Slow-3 (EFT)–Slow-4 (SRT)	.27	.11
Slow-5 (EFT)–Slow-4 (SRT)	.08	.67
Slow-4 (EFT)–Slow-3 (SRT)	.38*	.023

* denotes uncorrected *p* < .05.

## 4. Discussion

The present study examined the measurement reproducibility of sustained attention over time and across tasks. Conventional measurements (e.g., RT-SD, RT-CV) and a new measurement (RT-ALFF) were applied to the EFT and SRT. Three interesting findings were obtained: 1) moderate to high levels of reliability were identified from RT-ALFF patterns (Slow-5/4/3) and from conventional measurements of RT-SD and RT-CV for both tasks; 2) RT-ALFF magnitudes were found to be greater for the SRT than for the EFT and significant interactions between tasks were found by frequency band and 3) frequency-dependent correlations of RT-ALFF between the EFT and SRT were observed during both visits.

### 4.1. Test-retest reliability of the measurements

The reproducibility of conventional measurements of reaction time (i.e., RT-mean, RT-SD, RT-CV) and error rates has been documented in previous studies [32, 33]. The RT-mean shows better levels of test-retest reliability than the RT-SD and RT-CV. In the present study, excellent levels of reliability for the RT-mean and moderate to high levels of reliability for the RT-SD and RT-CV were identified. These results are consistent with previous findings showing that the RT-mean is typically more reliable than the RT-SD and RT-CV [32, 33].

As for error rates, we found poor to fair levels of reliability for all error rates on both tasks in line with prior observations [34, 35]. Error rates are usually employed as indices of sustained attention capacity [1, 36, 37]. Our results are based on healthy college students. This could partly account for the low error rates found. The low error rates found may be attributable to random factors that resulted in lower levels of test-retest reliability.

RT-ALFF is a frequency-dependent measurement of sustained attention [16, 24, 38]. Thus, its test-retest reliability was examined in several sub-frequency bands of Slow-6/5/4/3. Moderate to high levels of RT-ALFF reliability were observed in the Slow-5/4/3. It has been suggested that increased RT-ALFF levels in the low frequency band may be an endophenotype of sustained attention deficits [10]. Our results further reveal the strong reproducibility of RT-ALFF in the frequency band ranging from 0.01Hz to 0.167Hz. These low frequency bands thus deserve further exploration to illustrate the role of frequency-dependent RT-ALFF in sustained attention.

The EFT and SRT showed slightly different levels of test-retest reliability for RT-ALFF in a frequency-dependent manner. ICCs of RT-SD, RT-CV and RT-ALFF for the whole frequency band of the EFT were found to be slightly better than those of the SRT. However, for RT-ALFF levels in sub-frequency bands, ICCs of RT-ALFF for the EFT in Slow-5 and Slow-4 (.53 and .56) were found to be slightly smaller than those of the SRT (.58 and .68), and for Slow-3 the ICC of EFT (.74) was found to be larger than that of the SRT (.40). These results suggest that the RT-ALFF of the SRT is slightly more reliable than that of the EFT in low frequency bands. It remains uncertain which frequency band is more relevant to sustained attention. More frequency-dependent analyses of RT-ALFF are needed.

Both the EFT and SRT exhibited poor RT-ALFF reliability levels in the Slow-6. Few studies have addressed RT-ALFF values of below 0.01 Hz, as fluctuations in this frequency band have been ascribed to low frequency drifts [13, 26]. For our data, as is the case for all tasks of comparable length, sampling in the Slow-6 frequency band was found to be insufficient. Each task was performed over nearly eight minutes, meaning that frequencies of below 0.01 Hz were sampled less than four times. Moreover, accidental events, e.g., noise disturbances, can affect RT-ALFF levels in this frequency band. Likely due to these factors, RT-ALFF levels in the Slow-6 showed poor levels of reliability for both tasks.

### 4.2. Different frequency structures between the two tasks

RT-ALFF values of the SRT for each sub-frequency band were found to be larger than those of the EFT. Furthermore, significant interactions were found between tasks by frequency band. RT-ALFF levels in Slow-5 presented the largest differences between the two tasks. These results denote the presence of different frequency structures between the two tasks. This notion is also supported by the test-retest reliability and “band-shift” correlation results. The cognitive demands of these two tasks are different. The EFT is a relatively complex task involving higher levels of cognitive demand. For the EFT, it is necessary to select the arrow orientation and to disregard the flanking arrow during the task procedure [39–41]. The SRT involves a very limited number of selection and inhibition processing tasks. Thus, low demands of cognitive processes may have generated higher magnitudes of RT-ALFF and hence may serve as a purer paradigm for sustained attention studies. However, existing evidence is not sufficient enough to draw definitive conclusions on.

### 4.3. Correlations and differences in measurements between tasks

We found significant correlations in RT-mean values between the EFT and SRT for the two visits. However, RT-SD, RT-CV, and error rate values have been considered as indices of sustained attention capacity [36, 37]. Our results for the three measurements of sustained attention show that only the RT-SD value of the second visit presents a significant correlation between the two tasks. However, this correlation is highly inconsistent with that of the first visit (*r* = .41 vs. *r* = .02).

As noted above, the ICCs of the two tasks were found to be slightly different in a frequency-dependent manner. We were therefore inspired to perform a “band-shift” correlation, i.e., the RT-ALFF correlation for a frequency band of one task relative to that of its neighboring frequency band for another task (Table 7). Interestingly, the Slow-4 RT-ALFF of the EFT was found to be significantly correlated with that of the SRT Slow-5 for both visits (similar *r* = .357 and .356). Although corresponding *p* values do no pass the Bonferroni correction test, our band-shift correlation results may suggest that the relatively lower frequency fluctuations of the SRT and relatively higher frequency fluctuations of the EFT reflect the presence of sustained attention. However, it should be noted that the highest frequency band RT-ALFF of the EFT may be a complex reflection of mental processing, i.e., sustained attention, inhibition, and selection, due to conflicting conditions (incongruent vs. congruent) of the EFT. This issue should be investigated further.

### 4.4. Limitations

This study presents at least three limitations. First, to the facilitate frequency analysis, we used a fixed ITI (3 s) for the EFT and SRT, rendering the predictive effects of the subjects stronger. Second, error rates of the present study were exceedingly low, which might be due to random effects. The index presented is therefore not appropriate for sustained attention. Third, a low sampling rate of 3-s ITI only supports a frequency analysis of up to 0.167 Hz. A faster sampling rate would benefit explorations of RT-ALFF in relatively higher frequency bands.

## 5. Conclusions

Both conventional measurements and RT-ALFF results generated strong levels of test-retest reliability. RT-ALFF levels exhibit a frequency-dependent correlation between the EFT and SRT, although related frequency structures differ. These results suggest that RT-ALFF is an appropriate measure of sustained attention fluctuations.

## Supporting information

S1 Table Differences in Eriksen flanker task (EFT) RT-mean, RT-SD and RT-CV values across the three conditions. (DOCX)
