# Supplementary material for: Reproducibility of frequency-dependent low frequency fluctuations in reaction time over time and across tasks
Source: PLoS One. 2017 Sep 14;12(9):e0184476. doi: 10.1371/journal.pone.0184476 (PMC5598978; doi:10.1371/journal.pone.0184476)
Supplement: S1 Table — (DOCX) [file pone.0184476.s001.docx]

**S1 Table. Differences in Eriksen flanker task (EFT) RT-mean, RT-SD and RT-CV values across the three conditions.**

|  | *F* | *df* | *p* | η_p_^2^ | Pairwise comparisons |
| --- | --- | --- | --- | --- | --- |
| **RT-mean** |  |  |  |  |  |
| Visit | 1.23 | 1,34 | .28 | 0.035 | I>N>C |
| Condition | 112.50 | 2,33 | <0.001 | .87 |  |
| Visit×Condition | 1.16 | 2,33 | .33 | .066 |  |
| **RT-SD** |  |  |  |  |  |
| Visit | .88 | 1,34 | .36 | .025 | I>C,N |
| Condition | 7.64 | 2,33 | .002 | .32 |  |
| Visit×Condition | .06 | 2,33 | .94 | .004 |  |
| **RT-CV** |  |  |  |  |  |
| Visit | .55 | 1,34 | .46 | .016 |  |
| Condition | 2.25 | 2,33 | .12 | .12 |  |
| Visit×Condition | .06 | 2,33 | .94 | .004 |  |

I: incongruent condition, N: neutral condition, C: congruent condition
